# Neurologic Injury Associated with Rewarming from Hypothermia: Is Mild Hypothermia on Bypass Better than Deep Hypothermic Circulatory Arrest?

**DOI:** 10.3389/fped.2016.00104

**Published:** 2016-09-28

**Authors:** Utpal S. Bhalala, Elumalai Appachi, Muhammad Ali Mumtaz

**Affiliations:** ^1^Children’s Hospital of San Antonio, Baylor College of Medicine, San Antonio, TX, USA

**Keywords:** hypothermia, cardiopulmonary bypass, congenital heart defects, children, neuroinjury

## Abstract

Many known risk factors for adverse cardiovascular and neurological outcomes in children with congenital heart defects (CHD) are not modifiable; however, the temperature and blood flow during cardiopulmonary bypass (CPB), are two risk factors, which may be altered in an attempt to improve long-term neurological outcomes. Deep hypothermic circulatory arrest, traditionally used for aortic arch repair, has been associated with short-term and long-term neurologic sequelae. Therefore, there is a rising interest in using moderate hypothermia with selective antegrade cerebral blood flow on CPB during aortic arch repair. Rewarming from moderate-to-deep hypothermia has been shown to be associated with neuronal injury, neuroinflammation, and loss of cerebrovascular autoregulation. A significantly lesser degree of rewarming is required following mild (33–35°C) hypothermia as compared with moderate (28–32°C), deep (21–27°C), and profound (less than 20°C) hypothermia. Therefore, we believe that mild hypothermia is associated with a lower risk of rewarming-induced neurologic injury. We hypothesize that mild hypothermia with selective antegrade cerebral perfusion during CPB for neonatal aortic arch repair would be associated with improved neurologic outcome.

## Introduction

Congenital heart disease (CHD) is a common cause of morbidity and mortality in children. Eight in 1,000 newborns are diagnosed with CHDs, and approximately two to three million individuals are living in the US with CHDs (1, 2). Childhood survival with CHD has greatly improved over the decades following the first attempts at surgical intervention. Morbidity remains significant in survivors and is related to low cardiac output syndrome (LCOS), end-organ dysfunction, and neurological injury. Postoperative neurological injury increases the duration and cost of hospitalization and decreases postoperative quality of life. Neurological impairment is also linked to increased morbidity and mortality. Long-term neurocognitive outcomes in children with congenital heart disease are determined by variety of prenatal, perinatal, and postnatal factors, and some of them are not related to the surgical strategy. Cardiopulmonary bypass (CPB) surgery is a potential risk factor, which is known to contribute to the neurologic injury in this patient population. Various causative mechanisms have been postulated for neurological injury after CPB. These include embolization of gaseous and particulate matter, cerebral hypoperfusion, and systemic inflammatory response to CPB (3, 4). Various strategies have been described to reduce or prevent the occurrence of post-CPB neurological injury. Temperature manipulation during CPB is an important variable that could affect neurological outcome after CPB. Traditionally, aortic arch anomalies are repaired using deep hypothermic (18°C) circulatory arrest (DHCA) to allow for a bloodless field and adequate repair (5). However, DHCA provides a limited time for a complex repair with the potential for significant neurologic injury (6). To minimize the cerebral ischemia during DHCA, selective antegrade cerebral perfusion (SACP) has been utilized, but the results are conflicting (7–9) (Table 1). Many children’s centers continue to advocate deep hypothermia at 18°C with selective antegrade cerebral blood flow, during neonatal aortic arch repair. However, rewarming from moderate-to-deep hypothermia is associated with loss of cerebrovascular autoregulation and neuronal injury (10–15). Hypothermic CPB is associated with abnormal cerebrovascular autoregulation that is worsened with rewarming (11). Similarly, impairment of cerebral vascular reactivity has been reported in patients with traumatic brain injury after rewarming from therapeutic hypothermia (16). Degree and rate of rewarming from hypothermia in swine model of hypoxic ischemic cardiac arrest has been associated with increased neuronal apoptosis (17). The underlying mechanism of increased vulnerability to neurologic injury during rewarming from hypothermia is unknown, but it may be related to inadvertent cerebral hyperthermia (18). Mild hypothermia for aortic arch repair in adults has been reported to be simple, safe, and effective method of organ protection (19). Therefore, some centers have adopted mild hypothermia with SACP as a standard of care during aortic arch repair in children. To date, there is no study of short-term and long-term outcomes following mild hypothermia (32–35°C) with SACP in children undergoing aortic arch repair.

**Table 1:** **Summary of randomized controlled trials (RCTs) comparing neurologic outcomes following deep hypothermic circulatory arrest (DHCA) and selective cerebral perfusion (SCP)**.

Study	Subjects	Surgery	DHCA		SCP		Neurologic outcomes		
			E	A	E	A	Short-term	Long-Term	Imaging
Goldberg et al. (20)	Infants	Norwood surgery	38	30	39	27	Not studied	No difference	Not studied
Algra et al. (8)	Neonates	Aortic arch repair	19	18	18	18	No difference	Not studied	No difference
Harrington et al. (21)	Adult	Aortic arch surgery	21	20	21	21	SCP better than DHCA	No difference	Not studied
Vuylsteke et al. (22)	Adults	Pulmonary endarterectomy	35	30	39	36	Not studied	No difference	Not studied
Myung et al. (23)	Neonatal piglets	CPB with DHCA ± SCP	12	8	9	8	SCP better than DHCA	Not studied	Not studied

*RCTs, randomized controlled trials; DHCA, deep hypothermic circulatory arrest; SCP, selective cerebral perfusion, E, enrolled; A, analyzed*.

## Background

Survival of neonates with complex congenital heart defects (CHDs) continues to improve; however, cardiovascular and neurodevelopmental outcomes in survivors remain a concern. Neurodevelopmental impairment is the most common morbidity affecting the quality of life of children with CHD (24–26). Children who require cardiac surgery during the neonatal period have a significantly higher incidence of academic delays, motor difficulties, behavioral problems, issues with visual–motor integration and executive planning, language problems, inattention, and hyperactivity. The need for special services is significantly increased compared with the general population, as they get older (24). In a long-term follow-up study of patients with hypoplastic left heart syndrome, 30% received special education, the median IQ was 86, and 18% had some mental retardation (26).

## Modifiable Risk Factors and Outcomes in Neonates Following Repair for CHDs

Many known risk factors for adverse cardiovascular and neurological outcomes in neonates with CHD are not modifiable. The temperature and perfusion during CPB are two risk factors, which may be altered to improve long-term neurological outcomes. The relationship between metabolic rate and temperature was first described over 60 years ago, and hypothermia has become the mainstay of cardiac and neurological organ protection during CPB (5, 25). The complexity of the surgery, age, clinical status, and surgeon’s preference, all, determine the level of hypothermia during CPB (25). Four levels of hypothermia have been described: mild (33–35°C), moderate (28–32°C), deep (21–27°C), and profound (less than 20°C) (27, 28). For correction of specific types of CHDs, CPB and DHCA are used to optimize surgical visualization and allow complex repairs in a bloodless field. DHCA is associated with delayed return of cerebral blood flow and metabolism and increased risk of seizures during postoperative period (25, 29). In a study where neonates were randomly assigned to either DHCA or deep hypothermic low blood flow on CPB during aortic arch repair, both strategies were associated with increased risk of neurodevelopmental disabilities (6). SACP allows residual cerebral metabolism, so antegrade cerebral perfusion with moderate hypothermia has become the preferred strategy for adult aortic arch surgery patients and has also been utilized in some centers for children (19, 24). But, the results of SACP with moderate-to-deep hypothermia during aortic arch repair have been conflicting (7–9) (Table 1). In short, neither moderate hypothermia nor deep hypothermia, despite introduction of selective cerebral perfusion (SCP), has been associated with favorable outcomes.

## Effects of Rewarming from Hypothermia

**Figure 1:**
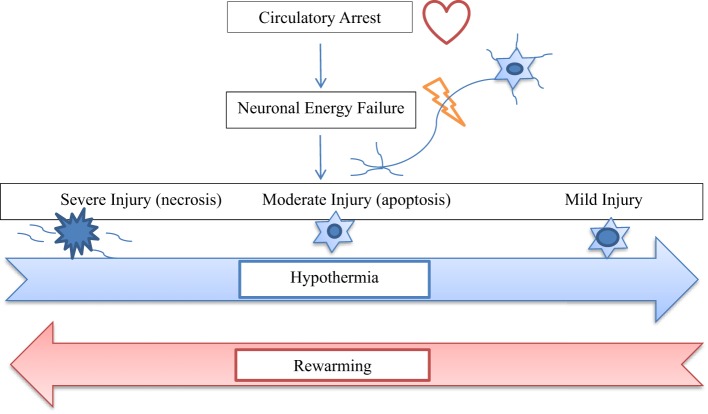
**The diagram shows relationship of hypothermia and rewarming with the spectrum of neuronal injury after circulatory arrest**. Hypothermia shifts cellular injury from necrosis to apoptosis to recovery, whereas, rewarming tends to worsen the degree of neuronal injury from apoptosis to necrosis.

**Figure 2:**
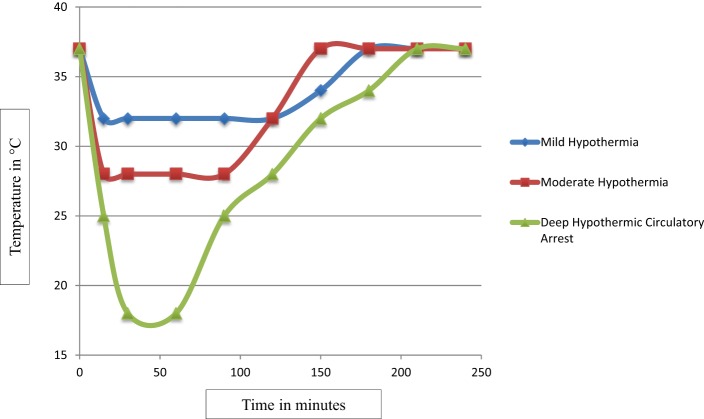
**The scatter plot of temperature changes during mild, moderate, and deep hypothermia and rewarming from each of mild, moderate, and deep hypothermia on CPB**. The degree and rate of rewarming from mild hypothermia differs significantly from the degree and rate of rewarming from moderate and deep hypothermia.

With growing evidence suggesting deleterious effects of prolonged circulatory arrest with profound hypothermia (24), moderate hypothermia with low flow CPB has been used to improve outcome. But, rewarming from moderate hypothermia has also been shown to be associated with loss of cerebral autoregulation and neuronal loss (10–15). Several animal studies suggest that even small increases in cerebral temperature (1–2°C) exacerbate ischemic neuronal injury (30–37). After moderate hypothermia, rewarming to 37°C was associated with a significant increase in average cerebrovascular pressure reactivity index, indicating temperature-dependent hyperemic derangement of cerebrovascular reactivity (16). Increased neuronal apoptosis has been demonstrated during rewarming from hypothermia in swine model of hypoxic ischemic cardiac arrest (17). In this study, at 29 h after hypoxia–ischemia, rewarmed piglets showed worse neuroapoptosis in motor cortex than did those that remained hypothermic. Neuroapoptosis in piriform cortex was also worse in hypoxic–ischemic, rewarmed piglets than in naive/sham piglets. Rate of rewarming was positively correlated with worse neuronal injury (17). In a similar study, piglets that received delayed hypothermia, followed by rewarming after hypoxia–ischemia, had significantly more apoptosis in the subcortical white matter than did normothermic piglets (38). The exact mechanism of deleterious effects of rewarming on cerebrovascular autoregulation and neuronal survival is not known. It is possible that physiologic and biochemical effects of rewarming from hypothermia are similar to those of fever or hyperthermia (Figure 1). Typically, fever or hyperthermia are associated with an increase in core body temperature by 2–10°C, whereas, restoration of normothermia from moderate-to-deep hypothermia on CPB involves 10–20°C of rewarming. Also, in most cases, such a significant degree of rewarming from moderate-to-deep hypothermia occurs over a short period of time on CPB (Figure 2). Due to significant concerns of degree and rate of rewarming from moderate-to-deep hypothermia, it is now time to readdress the ideal temperature strategy on CPB during neonatal aortic arch repair.

## Mild Hypothermia with Selective Antegrade Cerebral Perfusion During Neonatal Aortic Arch Repair

With mild hypothermia, the degree of rewarming required is reduced as compared with more aggressive hypothermia. As a result, rewarming-induced neuroinjury may be reduced with mild hypothermia as compared with moderate-to-deep hypothermia. Some centers have adopted mild hypothermia with SACP as a standard temperature strategy during the CPB in neonates undergoing aortic arch repair. The short-term outcomes of mild hypothermia with SACP during neonatal aortic arch repair are unknown. Postoperative end-organ dysfunction, such as LCOS, acute kidney injury, and acute neurologic injury, determine short-term outcomes after surgery for CHD. LCOS is one of the major causes of perioperative morbidity and mortality (39–41). Ischemia-reperfusion injury, inflammation, and postoperative cytokine balance play a central role in pathophysiology of end-organ dysfunction (42–44). Cytokine balance during cardiac surgery can be modified by pharmacologic and physical interventions (20, 44). Moderate hypothermia during CPB increases the synthesis of anti-inflammatory cytokines, blunts the release of pro-inflammatory cytokines, and provides organ protection (44). However, there is no information on the effects of mild hypothermia with SACP on pro-inflammatory and anti-inflammatory cytokines and end-organ function in neonates after aortic arch repair. Long-term outcomes of mild hypothermia with SACP during neonatal aortic arch repair have not been studied. Poor long-term neurodevelopmental outcome remains the major cause of morbidity and poor quality of life in the neonates surviving surgical repair of CHD. Evidence to support the deleterious effects of rewarming from hypothermia raises concerns about the neuroprotective effects of moderate or deep hypothermia, during CPB. Since the degree of rewarming differs significantly between moderate/deep hypothermia and mild hypothermia, it is conceivable that mild hypothermia offers better neuroprotection. As mild hypothermia during CPB is being used more widely as a standard temperature strategy, it is essential to learn about its long-term neurodevelopmental effects.

## Conclusion

Rewarming from moderate-to-deep hypothermia is associated with neuroinjury. Mild hypothermia is associated with only 3–5°C rewarming, whereas, moderate-to-deep hypothermia is associated with 10–20°C of rewarming to reach normothermia. Theoretically, mild hypothermia is associated with a significantly lower risk of rewarming-induced neuroinjury. It is conceivable that mild hypothermia with SACP is a rational strategy during CPB in neonates undergoing aortic arch repair. Short-term and long-term outcomes in children with CHDs following this newly adopted temperature strategy on CPB need a thorough evaluation, before it could be accepted widely.

## Author Contributions

All authors contributed equally to this work.

## Conflict of Interest Statement

The authors declare that the research was conducted in the absence of any commercial or financial relationships that could be construed as a potential conflict of interest.
